# Assessment of Operational Degradation of Pipeline Steels

**DOI:** 10.3390/ma14123247

**Published:** 2021-06-12

**Authors:** Hryhoriy Nykyforchyn, Olha Zvirko, Ihor Dzioba, Halyna Krechkovska, Myroslava Hredil, Oleksandr Tsyrulnyk, Oleksandra Student, Sebastian Lipiec, Robert Pala

**Affiliations:** 1 Department of Diagnostics of Materials Corrosion-Hydrogen Degradation, Karpenko Physico-Mechanical Institute of the NAS of Ukraine, 5 Naukova St., 79060 Lviv, Ukraine; olha.zvirko@gmail.com (O.Z.); krechkovskahalyna@gmail.com (H.K.); mysya.lviv@gmail.com (M.H.); otsyrulnyk@gmail.com (O.T.); oleksandrastudent1@gmail.com (O.S.); 2 Department of Machine Design, Faculty of Mechatronics and Mechanical Engineering, Kielce University of Technology, Av. 1000-an. of Polish State, 7, 25-314 Kielce, Poland; pkmid@tu.kielce.pl (I.D.); slipiec@tu.kielce.pl (S.L.); rpala@tu.kielce.pl (R.P.)

**Keywords:** transit pipeline steel, operational degradation, hydrogen, mechanical properties, electrochemical properties, microstructure, microfractography, numerical calculation method

## Abstract

This paper summarizes a series of the authors’ research in the field of assessing the operational degradation of oil and gas transit pipeline steels. Both mechanical and electrochemical properties of steels are deteriorated after operation, as is their resistance to environmentally-assisted cracking. The characteristics of resistance to brittle fracture and stress corrosion cracking decrease most intensively, which is associated with a development of in-bulk dissipated microdamages of the material. The most sensitive indicators of changes in the material’s state caused by degradation are impact toughness and fracture toughness by the J-integral method. The degradation degree of pipeline steels can also be evaluated nondestructively based on in-service changes in their polarization resistance and potential of the fracture surface. Attention is drawn to hydrogenation of a pipe wall from inside as a result of the electrochemical interaction of pipe metal with condensed moisture, which facilitates operational degradation of steel due to the combined action of operating stresses and hydrogen. The development of microdamages along steel texture was evidenced metallographically as a trend to the selective etching of boundaries between adjacent bands of ferrite and pearlite and fractographically by revealing brittle fracture elements on the fracture surfaces, namely delamination and cleavage, indicating the sites of cohesion weakening between ferrite and pearlite bands. The state of the X52 steel in its initial state and after use for 30 years was assessed based on the numerical simulation method.

## 1. Introduction

The main pipelines for the transportation of hydrocarbons, typically made of carbon steels, are long-term operated objects subjected to the action of corrosive environments. Therefore, their technical state should be periodically assessed. First, the state of the insulating coating is usually taken into account as well as the presence of operational macrodefects caused by mechanical and corrosive or corrosive-mechanical factors (cracks, corrosion pits, pipe wall thinning, etc.) [1,2]. However, for pipelines whose planned lifetime is already expired or close to expiring, it is necessary to evaluate thoroughly the possible degradation of the pipe metal, i.e., a loss of its initial physical and mechanical properties responsible for ensuring its operability. Among the mechanical properties are mainly the characteristics of strength and plasticity, brittle fracture resistance, and fatigue strength, including fracture toughness and fatigue crack growth resistance [1,2,3,4,5,6,7,8,9]. From the perspective of the effect of corrosive environments, it is worth determining the change in corrosion resistance and sensitivity of steels to stress corrosion cracking (SCC) and corrosion fatigue [1,4,10,11,12]. These are the main indicators of the metal state used most often to determine the operational loss of the pipeline serviceability and the increase in risk of unforeseen fractures of pipes in general.

Operational changes in the character and levels of the characteristics of mechanical fields in the areas of local stress and strain concentrations should also be considered. For an estimated conservative assessment of the strength of elements, it is sufficient to know the basic characteristics of the material yield strength, ultimate strength, and fracture toughness [13,14]. However, for a precise assessment, it is necessary to define the constitutive relationship of the material and perform various experimental testing and numerical calculations [15].

Considering the effect of corrosive environments on the reduction of serviceability of operated steels, it should be taken into account that degraded metal in general is more susceptible to such influences than as-received metal. This means that resistance to corrosion, SCC, and corrosion fatigue of degraded material is significantly lower than in the as-received state [11,12,16]. However, the other aspect of the problem is concerned with the influence of corrosive environments on the degradation of steel properties in the bulk of the material. Such an influence is possible in the case of the hydrogenating capability of working environments; the operational degradation of steels is then a result of the mutual effect of mechanical stresses and the hydrogen absorbed by metal. In general, a pipe wall could be hydrogenated from both external (due to breakdown of insulating coating) and internal surfaces. In the latter case, the reason is derived from the hydrogenating capability of transported hydrocarbons, particularly due to the presence of water and harmful admixtures. Thus, in this case, the essential part of the pipe wall could be hydrogenated as a result of electrochemical interaction of water condensed at the pipe internal surface with metal [16].

Analysing the operational degradation of pipe steels, special attention has been paid to the evolution of dissipated damages in the bulk of the material on nano- and microscales. This damaging significantly affects the properties of steels; primarily, it leads to a reduction of resistance to brittle fracture, worsening of corrosion, and hydrogen-assisted cracking characteristics [1,4,16]. The peculiarities of operational degradation are clearly revealed in this case: steel strength typically remains in the range corresponding to a given strength grade of pipeline steels. However, due to their low resistance to brittle fracture and enhanced susceptibility to corrosive-hydrogenating environments, they would be attributed to another, higher strength category.

A particular aspect of operational degradation of pipe steels is concerned with the metallographic research on steel structure changes. In contrast to high-temperature degradation [17], when microstructure changes are caused by high diffusion rates, as in the case of pipelines operated under ambient conditions, this factor is not so evident. However, the manifestation of the diffusion factor can be assumed at the nanostructure level. Indeed, a very low diffusivity of elements can be compensated by a tiny diffusion distance sufficient for the manifestation of the embrittlement effect of the local nanovolume of the metal (for instance, in the vicinity of grain boundaries [18]). This fact leads to a consideration of the degradation process on a nanoscale. If it is due to the initiation of dissipated damages in pipe steels, then its evolution should be traced starting from a nanoscale, not from a microscale.

One of the effective tools for the evaluation of degradation mechanisms in long-term operated steels is microfractographic research [19,20]. An in-service decrease of resistance to brittle fracture implies a corresponding change in fracture micromechanisms towards more brittle ones. Microfractography allows for identifying operational in-bulk damaging in pipe steels on micro- and nanoscales. 

It is also important to use nondestructive methods for evaluating the properties of a material, structural component, or structure to characterize its current technical state. There are various techniques to be used for this purpose. However, most of them only detect and size defects and damages. Nevertheless, several nondestructive methods have been developed and implemented, which enable evaluating mechanical properties of the material, such as indentation techniques [21], methods based on electrochemical correlations [22], etc.

The present paper aims to summarize the investigation results in various aspects of the operational degradation of pipe steels of gas mains, mainly performed in the frame of the NATO project G5055 [23,24,25] and under the scientific cooperation between the Karpenko Physico-Mechanical Institute of the NAS of Ukraine and Kielce University of Technology of Poland [26]. For these purposes, a number of existing mechanical, physical, and electrochemical methods suitable to degradation assessment have been used, and some new ones have been elaborated to evaluate the current state of operated metal.

## 2. Deterioration of Mechanical Properties

The object of this research is pipe steels of three strength grades (API 5L X52 and its equivalent 17H1S (0.2C-1.6Mn-0.6Si), API 5L X60, and API 5L X70), used for transit pipelines in Ukraine. The specimens were cut from reserve pipes (as-received state) and pipes after different times of operation. The dimensions of pipes (the outer diameter D and wall thickness t) are indicated in Table 1. The determined mechanical properties (yield strength σ_Y_, ultimate strength σ_UTS_, reduction in area RA, elongation at break, impact toughness KCV, fracture toughness J_0.2_–J integral for a crack propagation of 0.2 mm) of the tested operated steels (up to 51 years of operation) were compared with the corresponding characteristics of steels in the as-received state, which were in accordance with API 5L and DSTU ISO 3183:2017 standards (Table 1) [23]. A significant variation of mechanical properties inherent in such steels, even for the initial state of the metal, was taken into account, so the operational change in certain properties could be stated only by identifying a clear trend. Such trends have been established clearly for the characteristics of plasticity and resistance to brittle fracture (fracture toughness determined by the J-integral method and impact toughness). These characteristics essentially decrease; in particular, fracture toughness is the most sensitive regarding the assessment of the operational degradation of steels. Two indicators of the critical level of J-integral were determined: the start of fatigue precrack J_i_ and a 0.2 mm increment J_0.2_ according to the standard [27]. The value J_i_ characterizing the resistance to initiation of the static crack from the fatigue crack tip is less sensitive to steel operational degradation than the resistance to static crack propagation J_0.2_, which, in fact, depicts a part of the so-called R-curve. 

**Figure 1:**
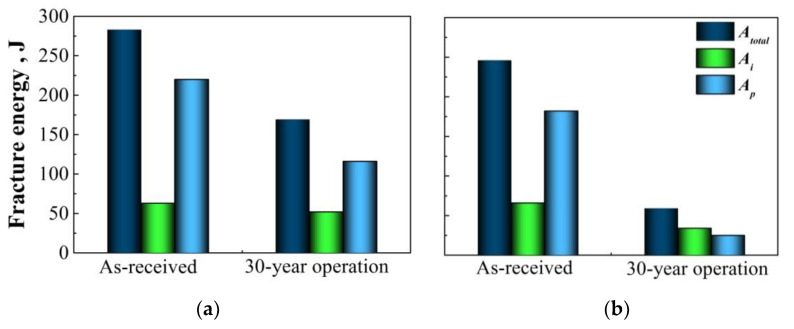
Total fracture energy A_total_ and its components of crack initiation A_i_ and crack propagation A_p_ during Charpy tests of X52 pipe steel at ambient (**a**) and −20 °C (**b**) temperatures.

Based on the revealed effect, the total fracture energy determined during the Charpy testing was separated into the components of the crack initiation energy A_i_ and the crack propagation energy A_p_ using instrumentalization of the testing facility. It was established for the case of X52 steel operated for 30 years (Figure 1) that almost the whole effect of operational decrease in impact toughness is due to the component A_p_. The effect is especially considerable under low-temperature test conditions (Figure 1b). This means that as in the case of fracture toughness (J-integral), the indicator of the resistance to crack propagation is especially sensitive to steel state and, therefore, more suitable for the assessment of steel degradation.

Pipes for transit pipelines are usually formed by rolling; therefore, a texture produced in steels after rolling in industrial processing conditions should be taken into account under the assessment of their operational degradation. In practice, this texture manifests itself by anisotropy in the mechanical properties of steel, as illustrated in Table 2 [28] for impact toughness measurements. The results showed essential differences in KCV values depending not only on the steel state (as-received vs. operated) but also on specimen orientation relative to the pipe axis. The highest KCV values were obtained for longitudinal specimens, regardless of the steel state. Impact toughness determined using tangential specimens is lower, and the difference is not considerable for the steels in the as-received state [29]; however, it became much more noticeable in the case of the operated steel. The lowest KCV values naturally are attributed to radial specimens where the fracture plane lies along texture fibres. This effect is in agreement with the results of the impact testing reported earlier [9] for long-term operated steels using specimens of different orientation relative to the pipe axis.

## 3. Changes in Electrochemical Behaviour

**Figure 2:**
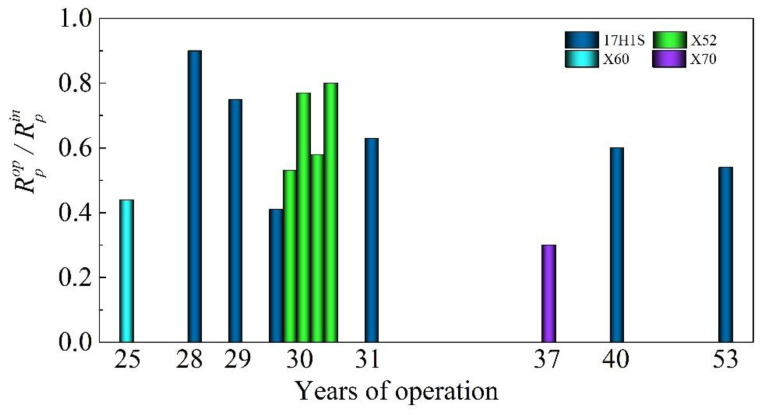
The changes in polarization resistance for 17H1S, X52, X60, and X70 pipe steels as a result of long-term operation *R^op^_p_* relative to the corresponding values in the as-received state *R^in^_p_*.

Electrochemical activation of pipe steels caused by operational degradation is manifested in the intensification of cathode and anode processes on the degraded steels [24]. This leads to an increase in corrosion current density, a decrease in polarization resistance, and the corrosion potential shift towards more negative values for the operated steels as compared with the steels in the as-received state (Table 3, Figure 2).

## 4. Microstructural and Fractographic Signs of Steel Degradation

**Figure 3:**
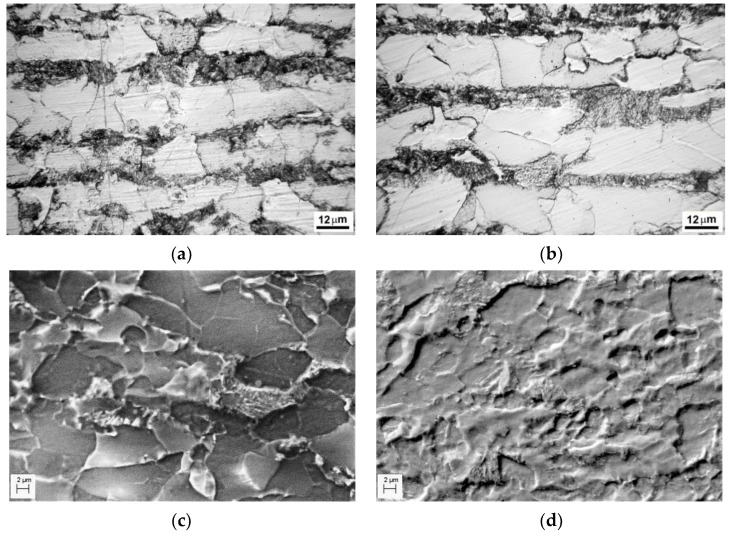
Microstructure of the 17H1S (**a**,**b**) and X70 (**c**,**d**) pipeline steels in the as-received state (**a**,**c**) and after operation for 30 (**b**) and 37 (**d**) years; at a distance of 3 mm from the outer surface of pipe in the transversal direction.

The investigated pipeline steels had ferrite–pearlite microstructures with a different texture. This texture is formed as a sequence of strips of ferrite and pearlite grains, which is typical for both as-received and operated steel states. It was the most clearly distinguished in the 17H1S steel structure [25]: polygonal ferrite grains were significantly varied in size (Figure 3a,b), and pearlite appeared in the form of layers, usually much thinner than ferrite ones and almost uninterrupted, up to 2 mm long, in the longitudinal direction, whereas in the short transversal direction, the length of pearlite layers was up to 150 microns only. The operated 17H1S steel was characterized by increasing sensitivity to etching of the steel microstructure in comparison with the unoperated steel (Figure 3a,b). Thus, the structure of the as-received steel was etched fairly uniformly, whereas some boundaries between adjacent grains of pearlite and ferrite were practically not manifested by etching of the exploited steel. Such an inhomogeneity of etching of the operated 17H1S steel microstructure was associated with its damaging on the microscale during long-term operation under a combined action of working stresses and corrosive media action with possible steel hydrogenation. The studies of the X60 and X70 pipeline steels revealed no evidence of susceptibility to increased etching of grain boundaries between ferrite and pearlite layers, which was explained by significant grinding of pearlite grains and mixture of cementite and ferrite inside them. Thus, the enhanced etching of grain boundaries was not observed in the X70 steel structure either in the as-received state (Figure 3c) or after long-term operation (Figure 3d) [25].

**Figure 4:**
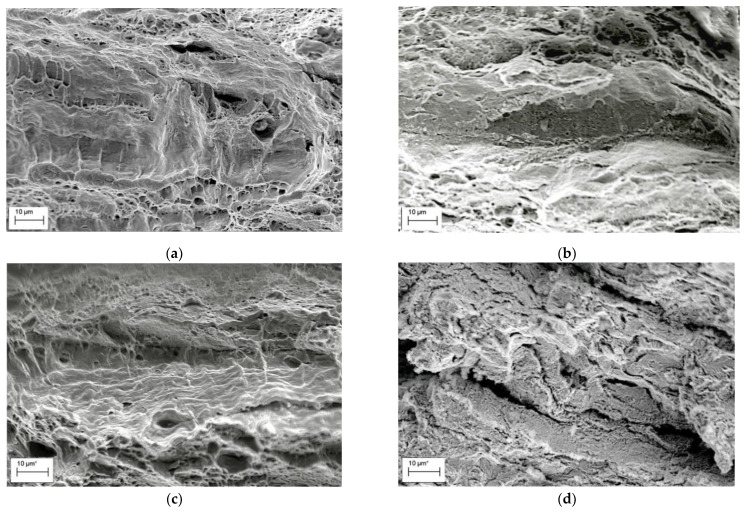
Fractograms of the specimens made of the 17H1S (**a**,**b**) and X70 (**c**,**d**) pipeline steels after operation for 30 (**a**,**b**) and 37 (**c**,**d**) years, fractured after tensile testing in air (**a**,**c**) and NS4 solution (**b**,**d**).

The specimen fracture under testing usually occurs along the crack path of minimum energy consumption (in other words, along the path with the maximum damaging). Therefore, operational damages in pipeline steels should be evident on the fracture surface of tested specimens [25]. Thus, delaminations were observed on the fracture surfaces of the operated 17H1S (Figure 4a,b) and X70 (Figure 4c,d) steels after slow strain rate tensile testing both in air and in NS4 solution, simulating a soil environment. Smooth surfaces with unidirectional elongated delaminations were observed on fracture surfaces of both steels after tensile testing in air (Figure 4a,c). The features of operational damages of steels were more clearly distinguished during tests in corrosive environments (Figure 4b,d); both quantity and length of delaminations on the fracture surface of the 17H1S steel were greater than on that of the X70 steel. This was considered as evidence of less severe damaging of the X70 steel during operation compared to the 17H1S steel. It should be noted that delaminations were sites for brittle fracture initiation by the mechanism of transgranular cleavage under tensile testing in a corrosive environment (Figure 4b,d). Moreover, such delaminations were observed not only near the specimen surface, which contacted with the environment under test, but also in the centre of the specimen cross section. Based on this, it was assumed that these delaminations already existed in the operated steel even before the tensile tests of specimens. Indeed, on the fracture surfaces of the same steels, in the as-received state, such fractographic features were not revealed. Therefore, it can be suggested that delaminations were formed during the operation of steels due to the accumulation of hydrogen along the boundaries of adjacent layers of ferrite and pearlite and hydrogen-induced adhesion weakening. Moreover, contrary to expectations, the X70 steel of a higher strength grade was characterised by less susceptibility to hydrogen embrittlement compared with the 17H1S steel of a lower strength grade. Such a peculiarity can be explained by the fragmentation of steel microstructure components and, consequently, less sensitivity to brittle fracture, including hydrogen embrittlement.

**Figure 5:**
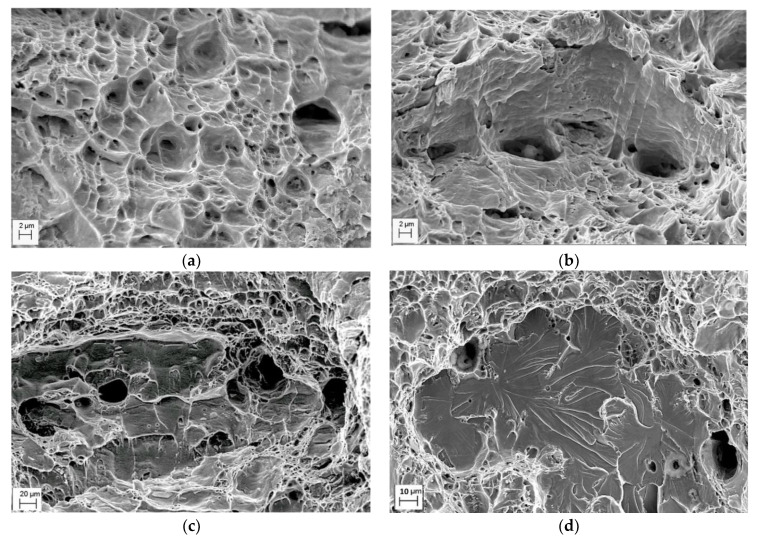
Typical fracture surfaces of pipeline steels after the Charpy testing: X70 (**a**,**b**) and 17H1S (**c**,**d**) in the as-received state (**a**) and after operation for 37 years (**a**), 30 (**c**), and 51 (**d**) years.

Analysing the fracture surface of specimens after impact toughness testing, certain fractographic features of steel degradation can be also distinguished [25]. Thus, in contrast to the as-received X70 steel specimen showing ductile fracture only (Figure 5a), some brittle fragments on the ductile fracture surface of the operated X70 steel specimens were found (Figure 5b). Delaminations of various lengths with pores in their depth were observed on the fracture surface of the operated X70 and 17H1S steel specimens (Figure 5b,c). Bridges between the delaminations were fractured by the dimple mechanism, which is typical for unoperated steels. Furthermore, some rounded fragments of cleavage (Figure 5d) were observed on the fracture surface of the 17H1S steel operated for 51 years, which was characterised by the highest degradation degree among the studied steels based on the assessment of operational decrease in impact toughness (Table 1). Therefore, delaminations and transgranular cleavage areas (Figure 5b–d) were considered as the features of in-service degradation of the pipeline steels. The development of delaminations (Figure 5b,c) and cleavage nucleation (Figure 5d) in operated steels was associated with hydrogen influence on steel degradation during long-term operation. The observed features are in a good agreement with other studies [9,19] where similar fractographic signs of embrittlement were detected for pipeline steels after operation or hydrogenation.

## 5. The Stress–Strain Analysis of Materials Condition Using the Numerical Simulation Method

The methods based on numerical simulation allows us to supplement and significantly deepen the strength analyses of the tested elements. Component load simulations carried out with numerical methods provide additional possibilities of obtaining information about the distribution of the mechanical fields inside them. This is very important in cases where there are crack-like defects. In order to obtain reliable results from numerical calculations, it is necessary to correctly define the constitutive relationship of the material and perform numerical modelling of the tested element.

**Figure 6:**
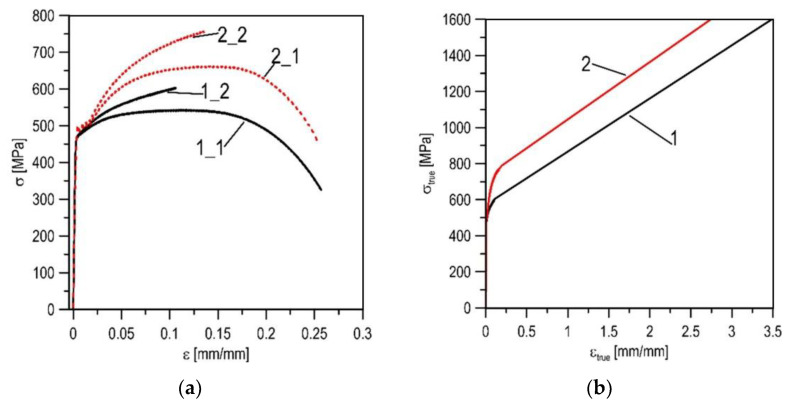
The stress–strain graphs of the X52 steel in as-received state (1) and after 30 years of service (2): (**a**)—the nominal curves (1_1, 2_1) and true curves for section of uniform tension (1_2, 2_2); (**b**) —the full true stress–strain dependences.

This chapter presents the results of the X52 steel state analysis obtained on the basis of numerical tests. The X52 steel in the initial state (as-received) and after 30 years of operation (used) were compared. In order to obtain high levels of stress and strain concentration in the material, specimens with a crack of a single edge notched beam (SENB) type were used in the numerical research. Defining the constitutive dependence of the material, the relationship between the true values of stresses and strains was carried out according to the calibration procedure presented in [30,31,32,33]. The graphs obtained on the basis of the uniaxial tensile test were the starting point for defining the constitutive relationship of the material (Figure 6a). Until the neck appears, during the uniform extension of the measuring section of the specimen, the actual values were calculated according to the well-known formulas: *ε*_true_ = ln (1 + *ε*_nom_); *σ*_true_ = *σ*_nom_ (1 + *ε*_nom_) (*ε*_nom_—nominal strain, *ε*_true_—true strain, *σ*_nom_—nominal stress, *σ*_true_—true stress). In the section of neck formation, the relationship of actual stresses and strains was presented with a linear function (Figure 6b). Critical values of stresses (*σ_C_*) and strains (*ε_C_*) for the tested materials were determined according to the procedure described in [26]: for the as-received X52 steel—*ε*_C_ = 3.50, *σ_C_ =* 1605 MPa; and after 30 years of operation—*ε*_C_ = 2.85, *σ_C_ =* 1630 MPa. 

**Figure 7:**
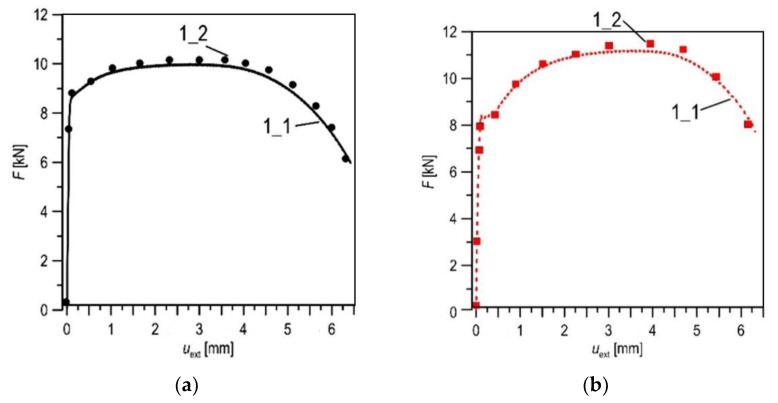
The comparison of the experimental (1_1) and numerical (1_2) *force-elongation* data for the X52 steel in the as-received state (**a**) and after 30 years of service (**b**).

**Figure 8:**
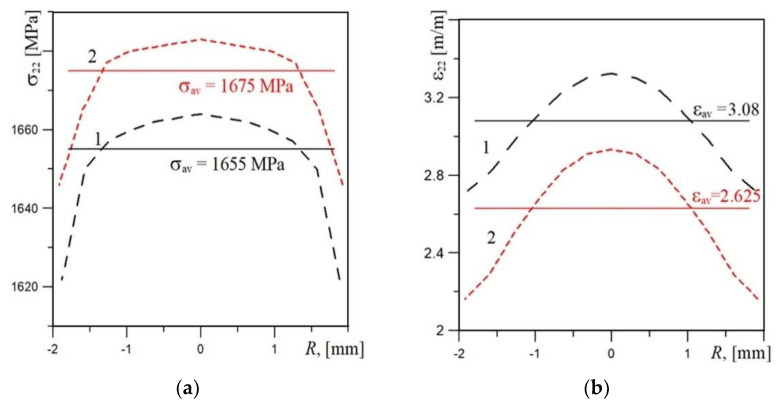
The stress (**a**) and strain (**b**) distributions obtained by numerical calculation (uniaxial tensile test specimen) for the X52 steel in the as-received state (1) and after 30 years of service (2), where *σ*_22_—stress in the tensile direction, *ε*_22_—strain in the tensile direction, *σ*_av_—the mean value of stress *σ*_22_, *ε*_av_—the mean value of strain *ε*_22_, *R*—the distance from specimen axis.

The correctness of the defined constitutive relationships was verified on the basis of the agreement of the experimentally recorded and numerically calculated *force*-*elongation* diagrams of the uniaxial tensile specimens (Figure 7). Another correctness criterion was the comparison of the numerically calculated stresses and strains at the moment of specimen failure with the critical values. The calculated stress and strain distributions in the axial cross-section of the specimens are shown in the graphs (Figure 8), and their values are similar to those designated as critical. The values of diameters of the specimens obtained according to measurements and calculation in the fracture moment are also similar.

**Figure 9:**
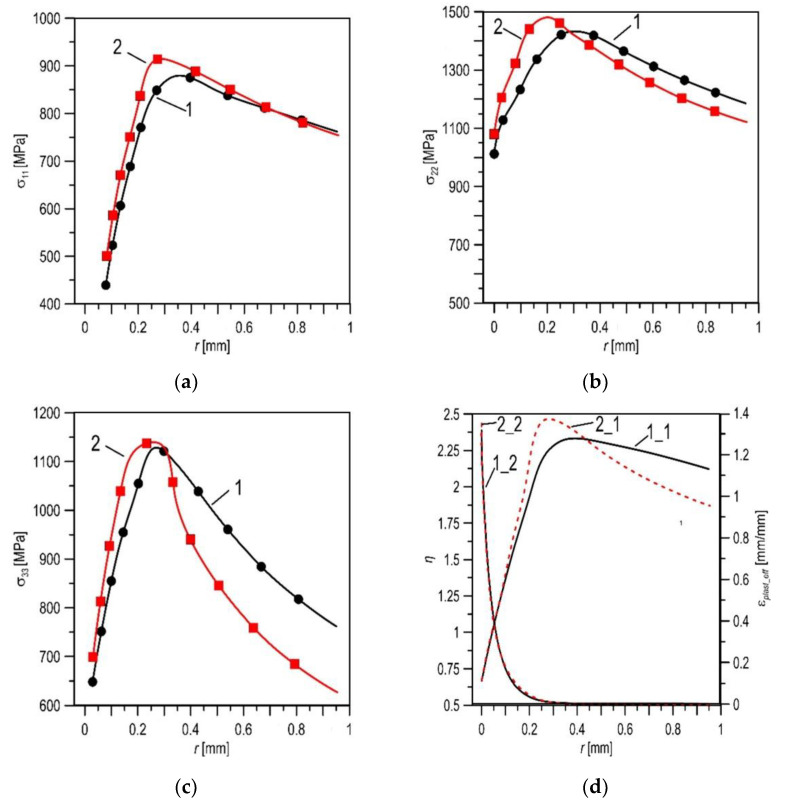
The distributions of stress components (**a**–**c**) of strain *e*_pl_eff_ and coefficient of triaxiality stress *η* (**d**) at load at *Du* = 1.2 mm: (**a**–**c**)—for the X52 steel in the as-received state, denoted as 1; and after 30 years of service, 2; (**d**)—for *η* (1_1), (2_1) and for *e*_pl_eff_ (1_2), (2_2), where *σ*_11_—stress in direction of crack growth, *σ*_22_—stress in the direction perpendicular to the crack plane, *σ*_33_—stress in the direction of the specimen thickness, *r*—distance from the crack tip.

In the next stage of the research, the stress and strain distributions were determined in front of the crack in the SENB specimens. A predefined constitutive relationship was introduced into the numerical model of the SENB specimens, and a load simulation was performed. The obtained distributions of stress and strain components and the triaxiality stress coefficient *η* (where *η = s*_eff_/*s*_m_ [31,32,33]), depending on the distance from the crack tip *r*, are presented in Table 4 and Figure 9. The distributions presented in Figure 9 are selected for the load that corresponds to the specimen deflection of *Du* = 1.2 mm.

The analysis of the results obtained by means of numerical calculations shows that in the X52 steel after 30 years of operation, the probability of a brittle fracture mechanism has increased significantly compared to that in the initial state. This is indicated by a number of signs. The maximum values of the stress components and the triaxiality stress coefficient *η* are higher for the steel after 30 years of service compared to the material in the as-received state. Additionally, for the exploited material, these maxima are located closer to the crack tip. Such a change in the location of the maximum values of the characteristics indicates that the material condition is approaching the area of brittle fracture [15,34,35]. This suggestion is indirectly confirmed by the analysis of the fracture surfaces of the specimens tested on impact strength; namely, local areas of cleavage cracking are revealed in the material after long-term usage (Figure 5). 

## 6. Sensitivity of Different Indicators of Metal State to Operational Degradation of Steels

As follows from the above, among the mechanical parameters suitable for the assessment of the operational degradation of pipe steels, the characteristics of resistance to brittle fracture, impact strength, and fracture toughness are the most sensitive. Concerning the plasticity parameters relative elongation and reduction in area, they should be distinguished separately if damaging develops in the material. Damage formation can be the result of either long-term operation or the specimen’s loading during mechanical testing. However, regardless of the nature of the damages, their opening contributes additionally to the elongation of the specimen and artificially increases the plasticity parameter. The phenomenon of a nominal increase in elongation of metal as a result of its operation, despite its actual embrittlement, has been revealed for the first time in [36], where the authors estimated the weld metal degradation of a steam pipeline operated on a thermal power plant. It was considered to be a peculiarity of the operational degradation of the material. Later this phenomenon was also confirmed concerning the X52 pipeline steel [37]. Thus, in some cases, it is even possible to obtain the increase in elongation relative to the metal in the as-received state. In such a case of operational increasing of elongation , preference should be given to the reduction in area, despite it not being included, as a rule, in regulatory documents on the mechanical properties of pipeline steels.

The diagrams “true stress–true strain” are proposed to be used for the evaluation of the operational degradation of the X52 pipe steel, since the true plastic strain value of material was sensitive to its degradation [26]. It is shown that the plastic deformation value estimated by the proposed plasticity indicator for the operated metal on the mentioned section of the stress–strain curve is considerably lower compared to the metal in the as-received state, despite the absence of noticeable differences in the reduction in area for these two metal states. Since plastic deformation on the decaying part of the curve is limited by the stage of damage formation in the neck, it is supposed that this plasticity indicator should depend on the resistance to growth and coalescence of defects formed in the specimen cross-section perpendicular to the applied load, and thereby will characterize, to some extent, the resistance of the material to crack propagation, that is, its fracture toughness. This assumption is in good agreement with the above mentioned special sensitivity of fracture toughness as the indicator of the material state in the assessment of its operational degradation.

It has been revealed [3,11,23] that using the fatigue crack growth rate has its own features in the assessment of operational degradation. It is well known that the Paris region of the fatigue crack growth curve is insensitive to structural changes in steels and thus to their operational degradation. In this case, the threshold value of the stress intensity factor could be used, taking into account the crack closure effect ΔK_th eff_, which is typical for crack growth in the near-threshold region. Therefore, the fatigue crack growth resistance of the steel in the as-received and operated state should be compared, instead, for the near-threshold section of the fatigue crack growth curve, taking into account the crack closure effect.

It is worth noting that the tests in corrosive environments may increase the sensitivity of the middle (Paris) part of the fatigue crack growth curve to the operational degradation of steels. This can be explained by the manifestation of steel susceptibility to SCC just at that specific part, which can lead to the leap of fatigue crack growth. Therefore, it is important to perform fatigue tests under a reduced frequency of cyclic loading under which the effect of the environment is especially noticeable. It should be also noted that hydrogen-induced degradation has a time-dependent nature, and a lower frequency leads to more severe degradation of the fatigue crack growth [19].

Concerning the sensitivity of SCC parameters to operational degradation, we can summarize based on the obtained results [37] that SSRT using precracked specimens is more efficient compared with using smooth ones. Evidently, this case also confirms the general trend: that the stage of crack growth propagation in steel is more sensitive to operational degradation than the crack initiation stage.

**Figure 10:**
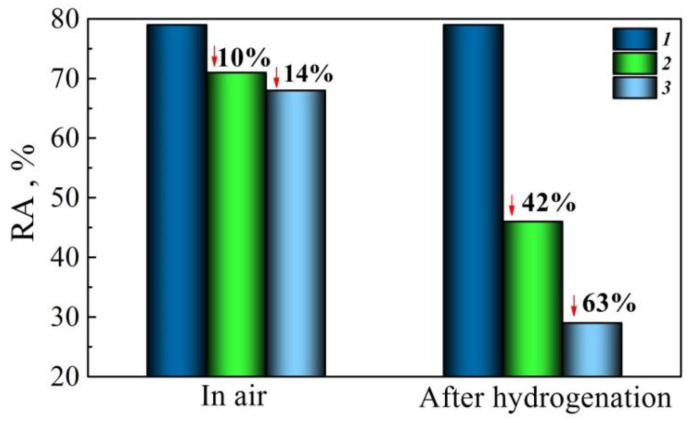
Changes in reduction in area RA for the 17H1S steel under the tensile tests: 1—as-received steel; 2, 3—operated for 30 and 40 years, respectively.

Another possibility exists of enhancing the sensitivity of certain parameters under the operational degradation assessment [38]. For instance, standard plasticity parameters are not sensitive enough; however, this drawback can be eliminated by preliminary hydrogenation of the test specimens. The presence of hydrogen as the factor of embrittlement should highlight the differences between different steel states since it is expected that the plasticity of degraded metal drops sharply under hydrogen action. In particular, the operation of the 17H1S pipe steel led to susceptibility to hydrogen embrittlement after its preliminary hydrogen charging, even for unloaded metal, and was more intensive for longer-operated steel (Figure 10). It can be noted that steel plasticity decreased as a result of operation by 14% if measured in air, whereas the effect rose to 63% after steel hydrogenation.

**Figure 11:**
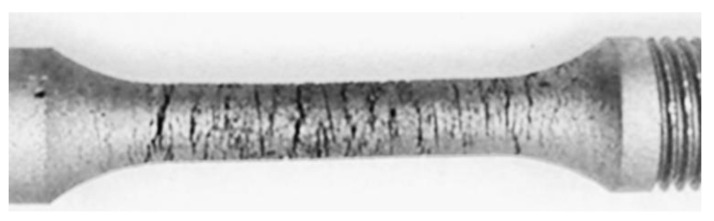
Smooth cylindrical specimen of a diameter of 5 mm after the combined action of tensile stress and hydrogen charging. Scale 1:2.

Thus, the hydrogenation of the specimens just before tensile tests in air leads to an enhancement of the susceptibility of the reduction in area to steel degradation by more than four times. Such an approach is suitable for increasing the sensitivity of the plasticity parameter to steel degradation regardless of the steel’s operating conditions (with or without environmental action). Preliminary hydrogenation of the metal simply makes it possible for the difference in the material state to manifest more clearly. However, it should be taken into account that hydrogen intensifies the surface cracking of the specimen as a result of its loading (as illustrated in Figure 11), and this contributes to overall elongation. Thus, this is the reduction in the area that is suitable for assessing steel plasticity, similar as in the case described above for nonhydrogenated specimens.

**Figure 12:**
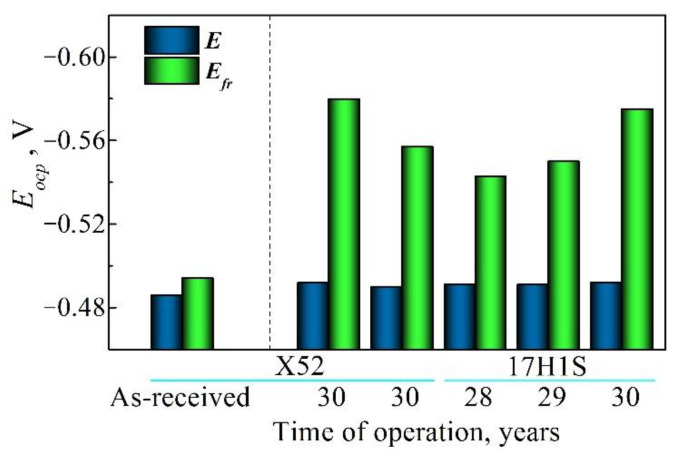
Open-circuit potentials E_ocp_ of the polished surface E and the fracture surface E_fr_ of the steel specimens made of the as-received and postoperated X52 and 17H1S steels (measured in 0.3% NaCl solution).

Some electrochemical methods have been proposed to estimate the operational degradation of steel since some electrochemical parameters revealed a sensitivity to in-service changes in material [22,24]. These include, first, polarization resistance and corrosion current density, whereas corrosion potential is not sensitive enough. Evidently, the reason is found in the electrochemical response of these parameters to operational defectiveness (damaging), which the real area of exposed surface depends on. However, regardless of the nature of the electrochemical response, electrochemical methods have the prospect of being used as nondestructive testing methods for assessing the current state of the operated metal. Within the frame of using the electrochemical approaches for such purposes, it is worth underlining the possibilities of the characterization of operated material by the electrochemical analysis of the fracture surfaces of the steel specimens tested to evaluate the mechanical properties sensitive to operational degradation [22,24]. This research has been performed using the fracture surfaces of impact specimens after the Charpy testing. In-service embrittlement of ferrite–pearlite pipe steels, which leads to their brittle fracture, was assumed to be associated with the precipitation of carbides of nano sizes at the grain boundaries and/or defects inside the grains over the course of long-term operation. Since fracture typically occurs through the weakest places in the material, the fracture surface of the specimens after the Charpy testing should be enriched with carbon compounds (obviously, carbide-type). It has been proven [22,24] that electrochemical parameters are sensitive enough to electrochemical microheterogeneity of the steel surface, including different carbon/carbide content. Indeed, a significant difference was observed between open-circuit potentials of the fracture surface (indicating brittle fracture) and the polished surface for the operated X52 and 17H1S steels (Figure 12). A shift of the potential towards more negative values for operated steels can be explained by an increased content of carbon compounds at the fracture surface as a result of steel in-service degradation [22]. As derived from Figure 5, open-circuit potential as an indicator of material state is sensitive enough to the in-service degradation of operated pipeline steels and can be used as an informative parameter for the evaluation of the current state of pipe steel.

## 7. Role of Hydrogen in Operational Degradation of Steels

It is known [38] that hydrogen accumulated during pipeline operation can be desorbed almost completely from the metal using the method of vacuum extraction under 600 °C. In the case of the X52 pipe steel, the total hydrogen content in the operated steel desorbed under 600 °C is two to three times higher than in the steel in the as-received state (Table 5) [37]. Moreover, higher hydrogen content is observed in the bottom sections of the operated pipes compared to their top sections. Such an increase in hydrogen content in the operated pipeline steel in comparison with that in the reserve pipe steel was also reported in [18], especially in the pipe section that failed under operation. 

**Figure 13:**
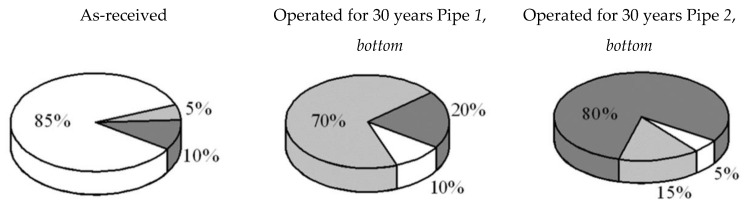
Hydrogen distribution in the X52 steel according to the results of vacuum extraction under different temperatures: 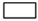 200 °C, 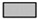 400 °C, 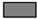 600 °C.

**Figure 14:**
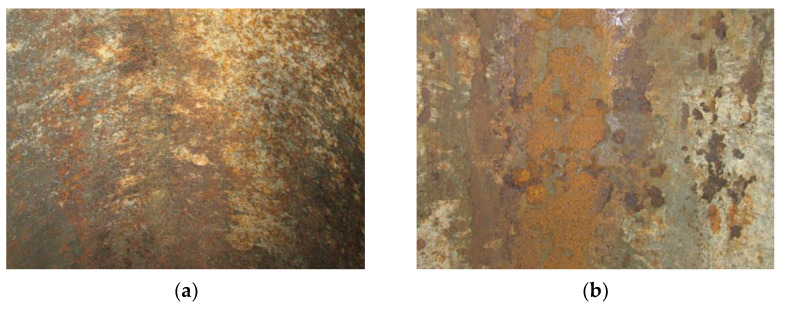
Corrosion defects at the inner surface of the pipeline made of the X52 steel: (**a**) top, (**b**) bottom. Scale 1:2.

It should be pointed out that hydrogen distribution between the fractions desorbed under different temperatures varies for the tested steels (Figure 13). Namely, hydrogen desorbs from the steel in the as-received state mainly under a low extraction temperature, leaving so-called reversible traps with a comparatively low binding capability. In contrast, most of the hydrogen desorbs from the operated steel under higher temperatures of extraction, indicating more intensive trapping of hydrogen inside the degraded metal, preferably at the grain boundaries, inclusions, and operational defects (irreversible traps). Among the two operated steels, the higher the amount of trapped “high-temperature” hydrogen, the worse the mechanical properties of the steel (see Table 1). Evidently, heightened hydrogen content in the vicinity of the pipe inner surfaces, especially in the bottom parts, is a result of the steel hydrogenation from the inside under corrosion as a result of the interaction of the steel with the corrosive-active components of the transported hydrocarbons. Extensive corrosion damages on the bottom of the operated pipe served as proof of intensive corrosion processes inside it (Figure 14). In contrast to pipelines transporting oil, where the residual water is always accumulated on the pipe bottom, in the case of gas pipelines, moisture can condense in different parts of the pipe inner surface depending on working and ambient conditions. Consequently, hydrogenation of the gas pipe is also possible over its entire inner surface. This is proven by the hydrogen-induced macrodelaminations revealed on various pipe sections, including its top part [9].

**Figure 15:**
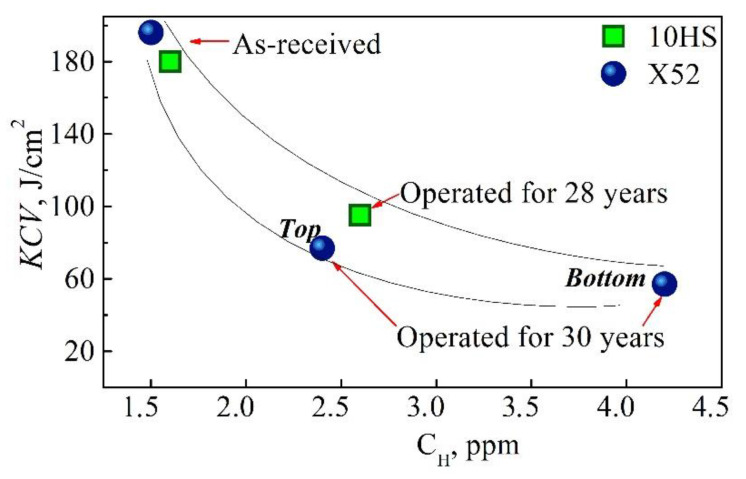
Concentration of hydrogen *C*_H_ measured by extraction method vs. impact toughness *KCV* for the 10HS and X52 pipe steels.

It should be also noted that the evaluation of hydrogen concentration through its desorption allows for measuring only residual hydrogen content since the tests were carried out after more than one year after the pipes had been put out of operation. That is why one should distinguish between the diffusible (atomic) hydrogen in the metal lattice and the residual hydrogen accumulated in defects as a result of hydrogen recombination into a molecular state. A heightened concentration of the residual hydrogen in operated steels is explained by intensive in-bulk microdamaging, which serves as traps for hydrogen. Damaging in turn causes a decrease, first, in brittle fracture resistance. Therefore, a relationship exists between hydrogen content in steels and their impact toughness, as derived from Figure 15, where lower values of impact toughness correspond to the operated metal with higher hydrogen concentrations, as compared to the as-received steel. It is possible that in-service worsening of brittle fracture resistance is concerned with not only in-bulk dissipated microdamaging, which serves as the stress multiconcentrator, but also the high pressure of hydrogen accumulated in defects as a factor of internal stresses at a microscale.

Concerning the role of the diffusible hydrogen absorbed by metal, its negative effect on the mechanical properties of steel can manifest themselves, first, by means of the facilitation of microdamage formation due to the hydrogen-assisted cracking mechanism and, as a consequence, reducing the resistance to brittle fracture. Its direct effect on impact toughness is most likely insignificant since there is not enough time to accumulate hydrogen of a high concentration in local volumes during the impact loading. Therefore, it is promising to assess the susceptibility of pipeline steels to hydrogen embrittlement by the J-integral parameter [39].

**Figure 16:**
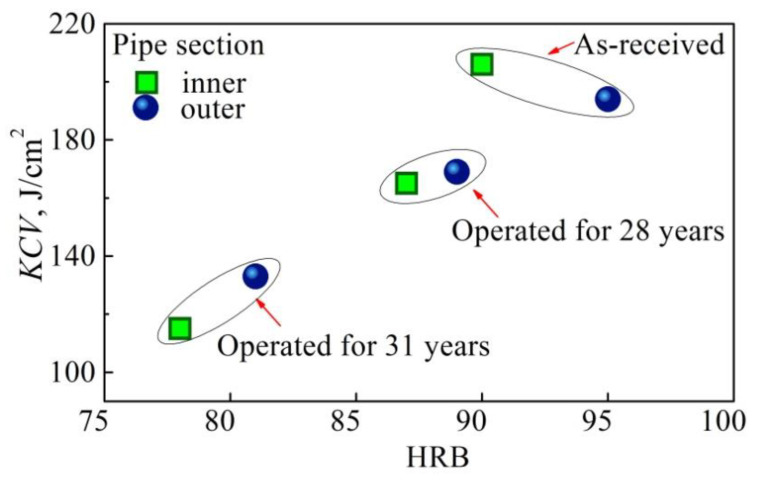
Relationship between hardness HRB and impact toughness KCV of the 17H1S steel.

The special role of transported media as a source of hydrogenation of the pipe wall and, as a consequence, a factor in the intensification of steel degradation is also illustrated in Figure 16. It can be seen that KCV values are slightly lower if Charpy specimens were cut in the vicinity of the inner surface of the operated pipe, whereas a similar influence of the location of specimen cutting on impact toughness values was not observed for unexploited steel. The metal in the as-received state instead exhibited the opposite tendency (slightly higher impact toughness near the inner surface of the pipe). A noticeable decrease of both hardness and impact toughness observed in the operated steel close to the pipe inner surface is proof of more intensive degradation of this part of the pipe.

Thus, hydrogenation of the pipe wall from the inside is an important factor in accelerating the operational degradation of the metal [16,23]. The mechanism of this acceleration consists in facilitating the occurrence of dissipated microdamages inside the metal, which, in turn, leads to a sharp decrease in the resistance to brittle fracture of pipeline steels.

## 8. Conclusions

1. The most significant factor in the loss of resistance to brittle fracture by long-term operated pipe steel is the development of dissipated damaging in the bulk of the material at nano and microscales. This damaging is also a reason for a decrease in fracture resistance under a corrosive-hydrogenating environment action. Hydrogenation of the pipe wall from its inner surface intensifies the operational degradation of steels.
2. Impact toughness and fracture toughness are the most sensitive indicators in the evaluation of the operational degradation of pipe steels. In addition, distinguishing the resistance to crack propagation as a component of the total fracture energy makes it possible to increase the sensitivity of these characteristics to steel degradation.
3. Steel degradation at the microscale was manifested by the nonuniform etching of boundaries between adjacent bands of ferrite and pearlite, indicating microdamage evolution along the steel texture as a result of the different permeability of hydrogen, accumulated during operation, in pearlite and ferrite. Hydrogen also promoted the occurrence of delaminations and cleavage fragments, which were revealed by fractographic analysis, and evidenced steel embrittlement due to its operational degradation.
4. Based on changes in the polarization resistance and surface fracture potential of steels caused by long-term service, their degradation degree can be evaluated.
5. The method of numerical simulations based on the precise definition and calibration of the constitutive relationship of the material allows for an accurate assessment of the material condition and an estimate of the strength of the elements, taking into account the stress concentration at crack-like defects.

## Figures and Tables

**Table 1:** Mechanical properties of pipe steels in the as-received state and after their long-term operation.

Pipe Steel	D, mm	t, mm	Time of Operation, Years		σ_Y_, MPa	σ_UTS_, Mpa	RA, %	Elongation, %	KCV, J/cm^2^	J_0.2_, kN/m
17H1S (X52 strength grade)	275	10	0 (as-received)		301	470	65.9	21.2	255	
	1220	12			380	624	72.0	23.9	129	
	1420	17			413	564	74.0	29.0	348	
	1420	17	30		368	541	55.3	26.3	175	
	1220	12	36		453	606	64.0	21.0	110	
	529	8	38		357	520	73.1	25.4	154	
	529	7	40		302	515	69.2	26.3	125	
	529	7	51		449	610	67.0	24.5	56/33 *	
X52	408	12	0		355	475	72.9	22.7	350	412
	275	12	30	Pipe 1	268	451	64.4	20.8	189	127
	275	10		Pipe 2	362	536	54.6	29.7	173	79
X60	529	14	0		510	592	81.9	23.2	342	
	1420	17	25		502	633	71.1	18.5	225	
X70	1420	17	0		521	615	73.4	22.3	277	
	1420	17	37		547	641	74.5	23.0	350/310 *	

Note. * KCV values obtained for transverse specimens relative to the pipe axis.

**Table 2:** Impact toughness of steels for different specimen orientation relative to the pipe axis.

Steel	State/Operation Time		KCV, J/cm^2^		
			Axial	Tangential	Radial
17H1S	As-received		152	129	–
	Operated	29 years	–	113	19
		36 years	–	84	38
X60	As-received		342	326	58
	Operated	25 years	326	214	37

**Table 3:** Basic electrochemical parameters of pipe steels measured in NS4 solution as a simulated soil solution.

Steel	Operation Time, Years	Corrosion Potential E_corr_, V	Corrosion Current Density i_corr_, μA/cm^2^	Tafel Constants, V	
				b_c_	b_a_
17H1S	0 (as-received)	–0.683	1.85	–0.090	0.062
	30	–0.687	4.20	–0.083	0.058
X60	0	–0.664	1.81	–0.090	0.063
	25	–0.696	3.86	–0.090	0.056
X70	0	–0.518	0.67	–0.089	0.061
	37	–0.642	2.24	–0.088	0.060

**Table 4:** Characteristic values of stress distributions at front of the crack tip.

D*u* [mm]	0.8 mm	1.0 mm	1.2 mm	1.4 mm	1.6 mm
max σ_22_ [MPa]	1445.61 ^a^	1436.32	1428.82	1423.21	1407.77
	1444.52 ^b^	1469.03	1480.68	1482.71	1493.61
*r* (max σ_22_) [mm]	0.240	0.287	0.337	0.317	0.424
	0.109	0.158	0.186	0.194	0.236
max *η*	2.408	2.341	2.311	2.303	2.303
	2.455	2.441	2.465	2.459	2.461
*r* (max *η*) [mm]	0.308	0.287	0.337	0.671	0.623
	0.244	0.293	0.273	0.326	0.455

^a^—data for the X52 steel in the as-received state; ^b^—data for the X52 steel after 30 years of operation.

**Table 5:** Concentration of hydrogen *C*_H_ in the X52 pipeline steel in the as-received state and after 30-year operation, measured by desorption at 600 °C.

Steel State	X52 (As-Received)	Operated Pipe 1				Operated Pipe 2			
		Top		Bottom		Top		Bottom	
		out	in	out	in	out	in	out	in
*C*_H_, ppm	1.5	1.2	0.7	1.5	1.4	1.7	2.8	3.1	5.1

## Data Availability

Data available on the request to the correspondence author.

## References

[1] Bolzon G., Boukharouba T., Gabetta G., Elboujdaini M., Mellas M. (2011). Integrity of pipelines transporting hydrocarbons. Proceedings of the NATO Advanced Research Workshop on Corrosion Protection of Pipelines Transporting Hydrocarbons.

[2] Witek M. (2021). Structural integrity of steel pipeline with clusters of corrosion defects. Materials.

[3] Vodenicharov S., Pluvinage G., Elwany M.H. (2008). Degradation of the physical and mechanical properties of pipeline material depending on exploitation term. Safety, Reliability and Risks Associated with Water, Oil and Gas Pipelines.

[4] Ohaeri E., Eduok U., Szpunar J. (2018). Hydrogen related degradation in pipeline steel: A review. Int. J. Hydrogen Energy.

[5] Filippov G.A., Livanova O.V., Chevskaya O.N., Shabalov I.P. (2013). Pipe steel degradation during operation and brittle failure resistance. Metallurgist.

[6] Maruschak P.O., Danyliuk I.M., Bishchak R.T., Vuherer T. (2014). Low temperature impact toughness of the main gas pipeline steel after long-term degradation. Cent. Eur. J. Eng..

[7] Kharchenko E.V., Klysz S., Palyukh V.M., Kunta O.E., Lenkovs’kyi T.M. (2017). Influence of the long-term operation of gas pipelines on the cyclic crack-growth resistance of 17G1S steel. Mater. Sci..

[8] Hutsaylyuk V., Maruschak P., Konovalenko I., Panin S., Bishchak R., Chausov M. (2019). Mechanical properties of gas main steels after long-term operation and peculiarities of their fracture surface morphology. Materials.

[9] Nykyforchyn H., Zvirko O., Tsyrulnyk O., Kret N. (2017). Analysis and mechanical properties characterization of operated gas main elbow with hydrogen assisted large-scale delamination. Eng. Fail. Anal..

[10] Quej-Ake L.M., Rivera-Olvera J.N., Domínguez-Aguilar Y.d.R., Avelino-Jiménez I.A., Garibay-Febles V., Zapata-Peñasco I. (2020). Analysis of the physicochemical, mechanical, and electrochemical parameters and their impact on the internal and external SCC of carbon steel pipelines. Materials.

[11] Krasovskii A.Y., Lokhman I.V., Orynyak I.V. (2012). Stress-corrosion failures of main pipelines. Strength Mater..

[12] Okipnyi I., Poberezhny L., Zapukhliak V., Hrytsanchuk A., Poberezhna L., Stanetsky A., Kravchenko V., Rybitskyi I. (2020). Impact of long-term operation on the reliability and durability of transit gas pipelines. Stroj. Casopis..

[13] Koçak M., Webster S., Janosch J.J., Ainsworth R.A., Koerc R. (2008). FITNET: Fitness-for-Service. Fracture-Fatique-Creep-Corrosion.

[14] Dzioba I., Tsyrulnik O.T. (2009). Analysis of the integrity of welded pipes of gas mains by the FITNET procedures. Mater. Sci..

[15] Dzioba I., Lipiec S. (2019). Fracture mechanisms of S355 steel—Experimental research, FEM simulation and SEM observation. Materials.

[16] Tsyrul’nyk O.T., Slobodyan Z.V., Zvirko O.I., Hredil M.I., Nykyforchyn H.M., Gabetta D. (2008). Influence of operation of Kh52 steel on corrosion processes in a model solution of gas condensate. Mater. Sci..

[17] Dzioba I. (2010). Properties of 13KHMF steel after operation and degradation under the laboratory conditions. Mater. Sci..

[18] Nechaev Y.S. (2008). Metallic materials for the hydrogen energy industry and main gas pipelines: Complex physical problems of aging, embrittlement, and failure. Uspekhi Fiz. Nauk..

[19] Alvaro A., Wan D., Olden V., Barnoush A. (2019). Hydrogen enhanced fatigue crack growth rates in a ferritic Fe-3 wt% Si alloy and a X70 pipeline steel. Eng. Fract. Mech..

[20] Maruschak P., Prentkovskis O., Bishchak R. (2016). Defectiveness of external and internal surfaces of the main oil and gas pipelines after long-term operation. J. Civ. Eng. Manag..

[21] Jang J.I., Choi Y., Lee Y.H., Kwon D. (2005). Instrumented microindentation studies on long-term aged materials: Work-hardening exponent and yield ratio as new degradation indicators. Mater. Sci. Eng. A.

[22] Nykyforchyn H., Tsyrulnyk O., Zvirko O., Krechkovska H. (2019). Non-destructive evaluation of brittle fracture resistance of operated gas pipeline steel using electrochemical fracture surface analysis. Eng. Fail. Anal..

[23] Nykyforchyn H., Bolzon G., Gabetta G., Nykyforchyn H. (2021). In-service degradation of pipeline steels. Degradation Assessment and Failure Prevention of Pipeline Systems. Lecture Notes in Civil Engineering.

[24] Zvirko O., Tsyrulnyk O., Bolzon G., Gabetta G., Nykyforchyn H. (2021). Non-destructive electrochemical evaluation of pipeline degradation. Degradation Assessment and Failure Prevention of Pipeline Systems. Lecture Notes in Civil Engineering.

[25] Krechkovska H., Hredil M., Student O., Bolzon G., Gabetta G., Nykyforchyn H. (2021). Structural and fractographic features of gas pipeline steel degradation. Degradation Assessment and Failure Prevention of Pipeline Systems. Lecture Notes in Civil Engineering.

[26] Dzioba I., Zvirko O., Lipiec S., Bolzon G., Gabetta G., Nykyforchyn H. (2021). Assessment of operational degradation of pipeline steel based on true stress–strain diagrams. Degradation Assessment and Failure Prevention of Pipeline Systems. Lecture Notes in Civil Engineering.

[27] Astm E. (1820). Standard Test Method for Measurement of Fracture Toughness ASTM. Annu. Book Stand..

[28] Zvirko O.I., Kret N.V., Tsyrulnyk O.T., Vengrynyuk T.P. (2018). Influence of textures of pipeline steels after operation on their brittle fracture resistance. Mater. Sci..

[29] Niu Y., Jia S., Liu Q., Tong S., Li B., Ren Y., Wang B. (2019). Influence of effective grain size on low temperature toughness of high-strength pipeline steel. Materials.

[30] Bai Y., Wierzbicki T. (2008). A new model of metal plasticity and fracture with pressure and lode dependence. Int. J. Plast..

[31] Wierzbicki T., Bao Y., Lee Y.-W., Bai Y. (2005). Calibration of seven fracture models. Int. J. Mech. Sci..

[32] Neimitz A., Gałkiewicz J., Lipiec S., Dzioba I. (2018). Estimation of the onset of crack growth in ductile materials. Materials.

[33] Neimitz A., Gałkiewicz J., Dzioba I. (2018). Calibration of constitutive equations under conditions of large strains and stress triaxiality. Archives of Civil and Engineering. Arch. Civ. Mech. Eng..

[34] Dzioba I., Gajewski M., Neimitz A. (2010). Studies of fracture processes in Cr-Mo-V ferritic steel with various types of microstructures. Int. J. Press. Vessel. Pip..

[35] Neimitz A., Gałkiewicz J., Dzioba I. (2010). The ductile to cleavage transition in ferritic Cr-Mo-V steel: A detailed microscopic and numerical analysis. Eng. Fract. Mech..

[36] Nykyforchyn H.M., Student O.Z., Markov A.D. (2007). Abnormal behavior of high-temperature degradation of the weld metal of low-alloy steel welded joints. Mater. Sci..

[37] Nykyforchyn H., Lunarska E., Tsyrulnyk O., Nikiforov K., Gabetta G. (2009). Effect of the long-term service of the gas pipeline on the properties of the ferrite–pearlite steel. Mater. Corros..

[38] Lunarska E., Polanskiy A. (2008). Kinetic measurements of hydrogen distribution between different states in exploited steels. Adv. Mater. Sci..

[39] Cabrini M., Sinigaglia E., Spinelli C., Tarenzi M., Testa C., Bolzoni F.M. (2019). hydrogen embrittlement evaluation of micro alloyed steels by means of J-Integral curve. Materials.

